# The psychology of discriminated ethnic minority individuals in the Netherlands; a Jungian perspective

**DOI:** 10.3389/fpsyg.2026.1764065

**Published:** 2026-07-02

**Authors:** Fatih Incekara

**Affiliations:** Erasmus University Medical Center, Rotterdam, Netherlands

**Keywords:** discrimination, Jung, mental health, persona, social identity

## Abstract

Discrimination is an influential structural determinant of mental health. Data show that at least two out of ten ethnic minority individuals in the Netherlands felt being discriminated against. Discrimination is not an incidental occurrence, but a structural recurring pattern within various parts of life especially during young adulthood. Ethnic discrimination is associated with a wide spectrum of psychological disturbances such as depression, anxiety and psychosis. This paper proposes a perspective through Swiss psychiatrist Carl Jung’s notion of *regressive restoration of the persona* as a conceptual framework to understand the psychological processes behind ethnic discrimination as a complex structural determinant undermining mental health. Jung conceptualized the persona as the social identity of the individual formed by society and argued that after adverse life events, one’s social identity can regress into a more limited social identity. The paper applies the concept of regressive restoration of the persona specifically to the context of discrimination among ethnic minority individuals in the Netherlands. I argue that discrimination alters the way ethnic minority individuals relate to society through a regressed social identity which forms during emerging adulthood. This Jungian conceptual framework aligns with theories such as the social defeat hypothesis and minority stress, which proposes that repeated experiences of discrimination increase the risk for psychological disturbances. This perspective points toward the relevance of future research on societal and culturally specific diagnosis and management strategies of psychological symptoms in discriminated ethnic minority individuals in the Netherlands especially during adolescence.

## Introduction

Discrimination is an influential structural determinant of mental health. Structural determinants refer to the broader social, political, economic and institutional arrangements that shape health and well-being (Hassan et al., 2023). From this perspective, discrimination should not be understood solely as an isolated, interpersonal experience, but as a recurring structural condition embedded in institutions and society at large. In the Dutch context, population-level data show that at least two in ten ethnic minority individuals in the Netherlands report having felt discriminated against in the previous year (CBS, 2024). Especially younger individuals felt being discriminated against more frequently. Race, nationality and religion are among the most frequently cited grounds, showing that discrimination is not merely an incidental occurrence, but a structural recurring pattern (CBS, 2024). Large cohort and survey studies further show that ethnic minority individuals and their descendants encounter discrimination across multiple social domains; work, education, public space and healthcare (Ikram et al., 2015; SCP, 2022).

There is substantial evidence that discrimination is associated with a wide range of psychological symptoms among ethnic minority individuals in the Netherlands, including depression (Ikram et al., 2015; Slotman et al., 2017), psychosis (Janssen et al., 2003; Veling et al., 2007; Francis-Crossley et al., 2025), psychological distress, anxiety, sleep problems and suicidal ideations (van Dijk et al., 2011; Willemen et al., 2023). Although the psychological impact of discrimination is well-known, discrimination remains a complex structural determinant. The social defeat hypothesis proposes that repeated experiences of discrimination increase the risk for psychological disturbances such as psychosis (Selten and Cantor-Graae, 2005, 2007; Selten et al., 2013), anhedonia and social withdrawal (Jaya et al., 2022), while the minority stress theory emphasises how stigmatized minority individuals are exposed to psychological distress. The psychological processes through which discrimination undermines mental health at the level of the individual need further conceptual elaboration.

Here, psychoanalytic concepts, and in particular Carl Gustav Jung’s notion of the *persona* and his description of the *regressive restoration of the persona*, may offer a beneficial conceptual framework to understand how ethnic discrimination affects the mental health of ethnic minority individuals (Jung, 1966). Jung conceptualized the persona as the social identity of the individual formed by society itself. He described how common adverse life events can threaten the social identity of the individual, potentially resulting in psychological disturbances due to a regressive, more defensive, withdrawn and neurotic, restoration of the persona (Jung, 1966).

This paper briefly summarizes epidemiological data on the association between ethnic discrimination and mental health among ethnic minority individuals in the Netherlands. It proposes Jung’s notion of *regressive restoration of the persona* as a conceptual framework for understanding the psychological processes through which ethnic discrimination undermines the mental health of ethnic minority individuals in the Netherlands.

### The epidemiology of discrimination

Population-based data indicate that discrimination is a common feature of life in the Netherlands, and that it is unequally distributed across groups. One report shows that 10.8% of residents aged 15 years and older, approximately 1.6 million people, reported having felt discriminated against in the previous 12 months (CBS, 2024). Among ethnic minority individuals (both people born in the Netherlands with at least one parent born abroad, or those born outside the Netherlands) approximately 20% reported discrimination, while 7.2% of native Dutch individuals reported discrimination (CBS, 2024). Race or skin color (38.5%) and nationality (33%) are among the most frequently mentioned reasons, followed by gender (28.3%), age (17.3%), religion or ideology (15.7%), sexual orientation (9%), political preference (8.5%) and disability or chronic illness (7.9%) (CBS, 2024).

A multi-ethnic cohort assessed ethnic discrimination in 6,546 participants. Frequent ethnic discrimination was reported by only 1.8% of ethnic Dutch participants, but by approximately 30% of participants in each of the non-Western minority groups (Ikram et al., 2015). This indicates that under identical institutional conditions, ethnic minority individuals are much more likely to report recurrent ethnic discrimination. A follow-up paper notes that 40–50% of ethnic minorities report some form of discrimination, especially in the labor market and public space (Ikram et al., 2016). A more recent report shows that established ethnic minority groups commonly report personal experiences of discrimination and frequently perceive the Netherlands as less hospitable toward people with a migration background (SCP, 2022). Second-generation and more highly educated ethnic minority individuals report more, not less, discrimination than first-generation and lower-educated peers (SCP, 2022). This phenomenon is known as the *integration paradox* in which greater social and linguistic participation coincides with greater exposure to ethnic discrimination (Schaeffer and Kas, 2023) and potentially to greater mental health deterioration (Bourque et al., 2011).

### The relationship between discrimination and mental health

A substantial body of data links discrimination to a spectrum of mental health outcomes, including depressive symptoms, psychosis, anxiety, sleep disorders, suicidal ideation and general psychological distress. A multi-ethnic cohort showed that both depressive symptoms and major depressive disorder were significantly more common in minority groups than in ethnic Dutch participants (Ikram et al., 2015). Ethnic discrimination accounted for a meaningful proportion of this excess burden. After adjustment for relevant covariates, each one-point increase in ethnic discrimination score was associated with odds ratios in the range of approximately 1.7–2.2 for depressive symptoms in minority groups (Ikram et al., 2015). The study suggested that up to a quarter of depression could theoretically be prevented if ethnic discrimination was absent (Ikram et al., 2015). Subsequent analyses found that discrimination was associated with higher depression scores across five minority groups, and that ethnic identity, religious involvement and dense co-ethnic social networks partly buffer this association (Ikram et al., 2016). Studies in younger populations converge on similar conclusions. In a school-based sample of young Turkish-Dutch and Moroccan-Dutch adults, it was reported that individual-level discrimination was significantly associated with elevated depressive symptoms indicating that discrimination exerts an independent effect on youth mental health (van Dijk et al., 2011). One report found that experiences of discrimination were more common among younger age groups (15–25 years: 14.3%; 25–45 years: 15.0%) than among older adults [45–65 years: 10.0%; ≥65 years: 4.3%, CBS (2024)]. The proportion of individuals with onset of any mental disorders before the age of 25 is 62.5% with peak age at 14.5 years (Solmi et al., 2022). This underscores the relevance of examining associations between discrimination and mental health outcomes during emerging adulthood.

Studies have also associated ethnic discrimination with psychotic disorders. A prospective study among the Dutch general population showed that perceived discrimination may induce delusional ideation and contribute to the high observed rates of psychotic disorder in exposed ethnic minority populations (Janssen et al., 2003). Veling et al. (2007) found that ethnic minority individuals that perceived high discrimination was associated with approximately a three- to fourfold increase in schizophrenia risk, situating discrimination as a population-level risk factor for psychosis. A recent large meta-analysis showed a significant association between discrimination and psychotic symptoms (adjusted OR = 1.77, 95%CI 1.26, 2.49) among 40,300 included participants (Francis-Crossley et al., 2025). By now, these findings have been reproduced in different parts of the world (Xavier et al., 2025). More recently, data from 17,053 participants across ethnic minority groups found that discrimination score was associated with suicidal ideation (Willemen et al., 2023). Finally, the CBS Safety Monitor captures a clear mental health signal: among respondents who reported discrimination, 28% attributed emotional or mental health problems to these experiences, 15% reported depressive symptoms, 11% sleep problems and around 8–9% anxiety or panic symptoms (CBS, 2024).

### Regressive restoration of the discriminated persona

The epidemiological and clinical data show that ethnic discrimination is a common structural determinant of mental health. However, the complexity of this structural determinant makes it difficult to pinpoint the processes by which discrimination undermines mental health on the level of the individual. Swiss psychiatrist Carl Jung conceptualized the persona as the social identity of the individual, formed out of society, through which the individual adopts and performs roles within society, “it is a compromise between individual and society as to what a man should appear to be. He takes a name, earns a title, exercises a function, he is this or that” (Jung, 1966). Jung argues that everyone experiences some kind of an adverse life event throughout life, ‘in all those careers where there has been some violent and destructive intervention of fate’ and he gives the example of a businessman going bankrupt (Jung, 1966, par 254). Such adverse life events can be embraced without causing permanent psychological disturbances within the individual, “If he does not allow himself to be discouraged by this depressing experience, but, undismayed, keeps his former daring, perhaps with a little salutary caution added, his wound will be healed without permanent injury” (Jung, 1966, par 254). However, the social identity gets affected if ‘on the other hand, he goes to pieces, abjures all further risks, and laboriously tries to patch up his social reputation within the confines of a much more limited personality, doing inferior work with the mentality of a scared child, in a post far below him, then, technically speaking, he will have restored his persona in a regressive way” (Jung, 1966, par 254).

To the best of my knowledge, this Jungian concept has never explicitly been applied to the context of ethnic discrimination. Discrimination is often not merely an incidental occurrence; it is a recurrent pattern of intentional “violent and destructive interventions” in lives of especially ethnic minority individuals. A regressive restoration of the social identity might be seen as the new compromise the ethnic minority individual is forced to make with the discriminating part of society. To the discriminated individual, the new, more limited social identity appears to be necessary to minimize future risk and to socially survive. Because discrimination occurs repeatedly rather than incidentally, the psychological “wound” Jung describes does not have the opportunity to heal. Healthy “little salutary caution,” as Jung proposes, may gradually develop into chronic suspicion and potentially to psychological disturbances such as hypervigilance, paranoia, psychosis, depression or social withdrawal. When the negotiation of identity between the individual and society is repeatedly undermined through ethnic discrimination, the resulting psychological impact on the social identity can potentially develop into psychological disturbances. It might be hypothesized that due to ethnic discrimination, ethnic minority individuals develop a regressive restoration of the persona as a defensive restriction of social identity. This, as a survival adaptation to societal exclusion or aggression, “which in the long run can be kept up only at the cost of neurotic sickliness” (Jung, 1966, par 259).

According to Jung, the regressive restoration of the persona is a self-alienation in favor of social demands. Jung proposes that the process of individuation, meaning self-realization through embracing individual uniqueness, may prevent regression of social identity and contribute to one’s social life; “individuation means precisely the better and more complete fulfilment of the collective qualities of the human being, since adequate consideration of the peculiarity of the individual is more conducive to a better social performance than when the peculiarity is neglected or suppressed”(Jung, 1966, par 267).

To illustrate the process of Jungian regressive restoration of the persona, consider a second-generation Turkish-Dutch university graduate entering the labor market. After repeated experiences of being rejected for internships despite strong qualifications, being asked whether she “really fits the team,” and being treated as culturally deviant in professional settings, she initially develops what Jung might call “salutary caution.” She prepares more carefully, monitors her speech, avoids discussing parts of her background and becomes alert to subtle signs of exclusion. Over time, however, this caution may become a more rigid defensive organization of the persona. She stops applying for positions that match her qualifications, avoids professional networks dominated by the ethnic majority, withdraws into a smaller social world and begins to interpret ambiguous social cues as signs of impending rejection. What initially functioned as adaptive self-protection may therefore become a regressive restoration of the persona: a constricted social identity organized around anticipated exclusion. Clinically, this may present as depressive symptoms, social withdrawal, hypervigilance or paranoid ideation.

As this case-example suggests, the negative effect of ethnic discrimination may particularly be relevant for emerging adults, for whom identity formation, educational transitions, entry into the labor market, intimate relationships and broader social integration are central developmental tasks. For ethnic minority emerging adults, these tasks unfold in a context in which social participation may increase exposure to discrimination. The so-called integration paradox is therefore especially important in this life phase: the younger adults participate in majority institutions, the more they may potentially encounter exclusion and discrimination. From a Jungian perspective, emerging adulthood can be understood as a formative period in which the persona is actively negotiated with society. Recurrent discrimination during this period may therefore have a particularly profound effect on the development of social identity. As meta-analyses specifically focusing on emerging adulthood consistently show, ethnic discrimination was indeed associated with poorer mental health during adolescence (Wang et al., 2026; Benner et al., 2018).

## Discussion

This paper briefly summarized data on the association between ethnic discrimination and mental health among ethnic minority individuals in the Netherlands. It showed that at least two out of ten ethnic minority individuals in the Netherlands felt being discriminated against. It also showed that discrimination is not an incidental occurrence, but a structural recurring pattern within various parts of life resulting in a wide spectrum of mental health disturbances among ethnic minority individuals. The paper also illustrates the negative impact of ethnic discrimination particularly during emerging adulthood. To understand the psychological processes behind ethnic discrimination as a complex structural determinant undermining mental health, the paper proposed Swiss psychiatrist Carl Jung’s notion of *regressive restoration of the persona* as a conceptual framework.

Jung conceptualized the persona as the social identity of the individual formed by society. In his work, he explains how adverse life events can influence the social identity of the individuals and provides an example of a businessman who becomes bankrupt (Jung, 1966). Jung argues that destructive hindrances experienced throughout one’s life can be dealt with by not getting discouraged and by continuing life with some extra caution. When one starts to avoid all future risks in life, “doing inferior work with the mentality of a scared child,” one will regress in a neurotic, more limited social identity (Jung, 1966, par. 259).

The paper puts the concept of regressive restoration of the persona specifically in the context of discrimination among ethnic minority individuals in the Netherlands. I argued that the widespread, exclusionary and recurrent nature of ethnic discrimination alters the way ethnic minority individuals relate to society through a regressive restored persona. Withdrawal into a narrowly defined in-group ethnic identity, or the quiet decision to stop applying for certain jobs, to avoid particular institutions, to “aim lower” than one’s qualifications would permit, or eventually to socially withdraw oneself can be seen as a regressive restoration of one’s social identity: a retreat to a smaller social field within which one’s value is less exposed to social threat. The person becomes, in Jung’s phrase, “smaller, more limited, more rationalistic” than before, resigned to a constricted version of oneself. The retreat into one’s own ethnic minority group can be seen as an adequate survival instinct, minimizing the risk of a threat from another group, however potentially at the cost of adapting a neurotic social attitude (Jung, 1966, p. 164). Aligning with this observation, a study found that the incidence of psychotic disorders was lower when one lived in a neighborhood with a corresponding high ethnic density (Veling et al., 2008). Comparable findings across multiple studies show that psychosis risk is substantially higher for ethnic minority individuals living in areas where they constitute a small minority, compared with areas with higher concentrations of people from similar ethnic backgrounds, which suggests that social exclusion or at least the impossibility to withdraw into one’s own in-group ethnic identity may be linked to a higher risk of developing psychological disturbances (Kirkbride et al., 2007; Boydell et al., 2001; Zammit et al., 2010). Prodromal and subsymptomatical questionnaires may be relevant to incorporate in future research on ethnic discrimination and mental health in order to illuminate the in-group identity experience of ethnic minority individuals.

This Jungian conceptual framework of ethnic discrimination may support the social defeat hypothesis, which proposes that repeated experiences of discrimination – being excluded, rejected, subordinated, or treated as an outsider – increase the risk for psychosis (Selten and Cantor-Graae, 2005, 2007; Selten et al., 2013). Similar findings have been reported between social defeat and negative symptoms such as amotivation, anhedonia and social withdrawal (Jaya et al., 2022). Whereas the social defeat model emphasizes the neurobiological consequences of long-term exclusion, Jung’s notion as proposed here may provide further understanding of the psychology of the discriminated ethnic minority individual and its relationship with society that follows such experiences. The Jungian interpretation also resonates with minority stress theory. Minority stress models describe how stigmatized minority individuals are exposed not only to external discriminatory events, but also to anticipatory stress, vigilance, concealment, internalized devaluation and chronic expectations of rejection (Frost and Meyer, 2023). The concept of regressive restoration of the persona may be read as a psychodynamic account of a similar process. Whereas minority stress theory conceptualizes the cumulative burden of chronic stressors, Jung’s notion of the persona helps specify how these stressors may reorganize the individual’s social identity. Together, these theories suggest that ethnic discrimination threatens the social identity of the ethnic minority individual which manifests itself as mental health deterioration such as depression, paranoia and psychosis.

For clinicians and researchers in cultural psychology, this framework implies that some forms of psychological disturbances among ethnic minority individuals – depression, anxiety, social withdrawal, suspicion, psychosis – may be understood as manifestations of a regressive restoration of the social identity, of which the latter is rooted in emerging adulthood. The notion suggests that the processes are understandable attempts to survive in a social environment where each step toward fuller participation potentially increases the risk of a recurrent painful discriminatory blow. This Jungian perspective highlights the relevance of future research on societal and culturally specific diagnosis and management strategies of psychological symptoms in discriminated ethnic minority individuals in the Netherlands.

## Data Availability

The original contributions presented in the study are included in the article/supplementary material, further inquiries can be directed to the corresponding author.

## References

[ref1] Benner A. D. Wang Y. Shen Y. Boyle A. E. Polk R. Cheng Y. P. (2018). Racial/ethnic discrimination and well-being during adolescence: a meta-analytic review. Am. Psychol. 73, 855–883. doi: 10.1037/amp0000204, 30024216 PMC6172152

[ref2] Bourque F. van der Ven E. Malla A. (2011). A meta-analysis of the risk for psychotic disorders among first- and second-generation immigrants. Psychol. Med. 41, 897–910. doi: 10.1017/S0033291710001406, 20663257

[ref3] Boydell J. van Os J. McKenzie K. Allardyce J. Goel R. McCreadie R. G. . (2001). Incidence of schizophrenia in ethnic minorities in London: ecological study into interactions with environment. BMJ 323, 1336–1338. doi: 10.1136/bmj.323.7325.1336, 11739218 PMC60671

[ref4] CBS (2024). Veiligheidsmonitor 2023: Ervaren discriminatie. Den Haag: CBS.

[ref5] Francis-Crossley I. Hudson G. Harris L. Onwumere J. Kirkbride J. B. (2025). The association between racism and psychosis: an umbrella review. PLOS Ment Health 2:e0000401. doi: 10.1371/journal.pmen.0000401, 41662051 PMC12798482

[ref6] Frost D. M. Meyer I. H. (2023). Minority stress theory: application, critique, and continued relevance. Curr. Opin. Psychol. 51:101579. doi: 10.1016/j.copsyc.2023.101579, 37270877 PMC10712335

[ref7] Hassan I. Chisty A. Bui T. (2023). “Structural and social determinants of health,” in Leading an Academic Medical Practice, eds. Lu L. B. Fortuna R. J. Noronha C. F. Sobel H. G. Tobin D. G. (Cham: Springer).

[ref8] Ikram U. Z. Snijder M. B. de Wit M. A. Schene A. H. Stronks K. Kunst A. E. (2016). Perceived ethnic discrimination and depressive symptoms: the buffering effects of ethnic identity, religion and ethnic social network. Soc. Psychiatry Psychiatr. Epidemiol. 51, 679–688. doi: 10.1007/s00127-016-1186-7, 26873614 PMC4846702

[ref9] Ikram U. Z. Snijder M. B. Fassaert T. J. L. Schene A. H. Kunst A. E. Stronks K. (2015). The contribution of perceived ethnic discrimination to the prevalence of depression. Eur. J. Pub. Health 25, 243–248. doi: 10.1093/eurpub/cku180, 25416918

[ref10] Janssen I. Hanssen M. Bak M. Bijl R. V. de Graaf R. Vollebergh W. . (2003). Discrimination and delusional ideation. Br. J. Psychiatry 182, 71–76. doi: 10.1192/bjp.182.1.71, 12509322

[ref11] Jaya E. S. Pillny M. Lincoln T. M. Riehle M. (2022). Does social defeat cause negative symptoms? A prospective study in a multi-national community sample. Compr. Psychiatry 113:152289. doi: 10.1016/j.comppsych.2021.152289, 34942483

[ref12] Jung C. G. (1966). Two Essays on Analytical Psychology. Collected Works, vol. 7. 2nd Edn Translated by R.F.C. Hull. London: Routledge & Kegan Paul.

[ref13] Kirkbride J. B. Morgan C. Fearon P. Dazzan P. Murray R. M. Jones P. B. (2007). Neighbourhood-level effects on psychoses: re-examining the role of context. Psychol. Med. 37, 1413–1425. doi: 10.1017/S0033291707000499, 17472758

[ref14] Schaeffer M. Kas J. (2023). The integration paradox: a review and meta-analysis of the complex relationship between integration and reports of discrimination. Int. Migr. Rev. 58, 1384–1409. doi: 10.1177/01979183231170809

[ref15] SCP (2022). Gevestigd, maar niet thuis. Eerste bevindingen uit de Survey Integratie Migranten (SIM 2020). Den Haag: SCP.

[ref16] Selten J. P. Cantor-Graae E. (2005). Social defeat: risk factor for schizophrenia? Br. J. Psychiatry 187, 101–102. doi: 10.1192/bjp.187.2.101, 16055818

[ref17] Selten J. P. Cantor-Graae E. (2007). Hypothesis: social defeat is a risk factor for schizophrenia? Br. J. Psychiatry Suppl. 51, s9–s12. doi: 10.1192/bjp.191.51.s9, 18055945

[ref18] Selten J. P. van der Ven E. Rutten B. P. Cantor-Graae E. (2013). The social defeat hypothesis of schizophrenia: an update. Schizophr. Bull. 39, 1180–1186. doi: 10.1093/schbul/sbt134, 24062592 PMC3796093

[ref19] Slotman A. Snijder M. B. Ikram U. Z. Schene A. H. Stevens G. W. J. M. (2017). The role of mastery in the relationship between perceived ethnic discrimination and depression: the HELIUS study. Cult. Divers. Ethn. Minor. Psychol. 23, 200–208. doi: 10.1037/cdp0000113, 27454888

[ref20] Solmi M. Radua J. Olivola M. Croce E. Soardo L. Salazar de Pablo G. . (2022). Age at onset of mental disorders worldwide: large-scale meta-analysis of 192 epidemiological studies. Mol. Psychiatry 27, 281–295. doi: 10.1038/s41380-021-01161-7, 34079068 PMC8960395

[ref21] van Dijk T. K. Agyemang C. de Wit M. Hosper K. (2011). The relationship between perceived discrimination and depressive symptoms among young Turkish-Dutch and Moroccan-Dutch. Eur. J. Pub. Health 21, 477–483. doi: 10.1093/eurpub/ckq093, 20621959

[ref22] Veling W. Selten J.-P. Susser E. Laan W. Mackenbach J. P. Hoek H. W. (2007). Discrimination and the incidence of psychotic disorders among ethnic minorities in the Netherlands. Int. J. Epidemiol. 36, 761–768. doi: 10.1093/ije/dym085, 17517810

[ref23] Veling W. Susser E. van Os J. Mackenbach J. P. Selten J. P. Hoek H. W. (2008). Ethnic density of neighborhoods and incidence of psychotic disorders among immigrants. Am. J. Psychiatry 165, 66–73. doi: 10.1176/appi.ajp.2007.07030423, 18086750

[ref24] Wang Y. Huang Q. Kim D. Liu J. Lin S. Zhang Y. . (2026). Systematic review and meta-analysis: racial-ethnic discrimination and young people’s mental health in intensive longitudinal studies. J. Am. Acad. Child Adolesc. Psychiatry 65, 652–668. doi: 10.1016/j.jaac.2025.06.007, 40541925

[ref25] Willemen F. E. M. Heuschen C. B. B. C. M. Zantvoord J. B. Galenkamp H. de Wit M. A. S. Zwinderman A. H. . (2023). Perceived ethnic discrimination, suicidal ideation and mastery in a multi-ethnic cohort: the HELIUS study. BJPsych Open 9:e21. doi: 10.1192/bjo.2022.640 36660955 PMC9885336

[ref26] Xavier S. M. Both A. van der Ven E. Ferrari M. Abdel-Baki A. van den Bogerd N. . (2025). Experiences and socio-environmental contexts in the lead-up to psychosis: a qualitative analysis of the narratives of persons with psychosis from different ethnic, racial and immigrant backgrounds. Front. Psych. 16:1602468. doi: 10.3389/fpsyt.2025.1602468, 41127217 PMC12537757

[ref27] Zammit S. Lewis G. Rasbash J. Dalman C. Gustafsson J. Allebeck P. (2010). Individuals, schools, and neighborhood: a multilevel longitudinal study of variation in incidence of psychotic disorders. Arch. Gen. Psychiatry 67, 914–922. doi: 10.1001/archgenpsychiatry.2010.101, 20819985

